# Order-Aware Energy Recycling for Backscatter-Aided Wireless-Powered Cooperative Communications

**DOI:** 10.3390/s26144357

**Published:** 2026-07-09

**Authors:** Yuan Zheng, Dongqing Li, Huan Wan

**Affiliations:** 1 School of Integrated Circuits, Shenzhen Polytechnic University, Shenzhen 518055, China; 2 School of Electronic and Communication Engineering, Shenzhen Polytechnic University, Shenzhen 518055, China; 3 Undergraduate School of Artificial Intelligence, Shenzhen Polytechnic University, Shenzhen 518055, China; 4 Shenzhen Key Laboratory of Media Security, Guangdong Provincial Key Laboratory of Intelligent Information Processing, Shenzhen University, Shenzhen 518060, China

**Keywords:** wireless-powered sensor networks, backscatter communication, cooperative communication, energy recycling, throughput fairness

## Abstract

In wireless-powered sensor networks, active uplink (UL) transmissions can support both sensed-data delivery and order-dependent RF energy recycling among energy-constrained devices. This paper studies a single hybrid access point (HAP) wireless-powered sensor network (WPSN), where one wireless device (WD) is selected as the relay, and the remaining WDs act as sources. The HAP first transfers wireless energy to all WDs in the downlink. Then, the source WDs reuse the incident wireless energy signal as a controllable carrier and deliver their information to the relay through passive backscatter communication. In the subsequent active uplink phase, the source WDs transmit according to an optimized order, while later scheduled sources recycle energy from the active signals transmitted by preceding sources. The source order therefore determines not only the transmission sequence but also the directed energy-recycling structure among source WDs, thereby reshaping the energy-causality constraints and the feasible transmit-energy region. The relay finally forwards the decoded source information to the HAP. To improve throughput fairness among sensor devices, a max-min throughput optimization problem is formulated by jointly designing the source transmission order, time allocation, and transmit-power allocation. For a given order, the continuous resource allocation problem is transformed into a convex problem through transmit-energy variables, and the optimal order is obtained by searching over all candidate orders. Numerical results show that the proposed scheme achieves higher minimum throughput than the three representative comparison schemes, demonstrating the benefit of jointly exploiting passive source-to-relay collection, relay-assisted forwarding, and order-aware energy recycling.

## 1. Introduction

The increasing density of low-power wireless devices (WDs) has made energy sustainability a central issue in Internet-of-Things (IoT) networks. Many IoT devices are expected to operate for long periods with limited battery capacity, while manual battery replacement becomes costly or even impractical in large-scale deployments. Radio-frequency (RF) energy harvesting provides a feasible way to support battery-limited wireless sensing and communication by converting received RF signals into usable electrical energy [1,2,3]. Wireless powered communication networks (WPCNs), also referred to as wireless-powered sensor networks (WPSNs) in sensing-oriented applications, exploit this idea by allowing WDs to harvest energy from a hybrid access point (HAP) in the downlink (DL) and then use the harvested energy for uplink (UL) wireless information transmission. The harvest-then-transmit protocol and the associated resource allocation design have been widely studied in WPCNs [4,5]. Recent studies have also applied intelligent optimization to energy-constrained and interference-limited wireless networks, such as energy-aware routing in underwater wireless sensor networks [6] and anti-interference design in heterogeneous UAV communications [7]. However, the same HAP-to-WD link governs both DL energy harvesting and UL data delivery. WDs with unfavorable HAP links harvest less energy but require more transmit energy, which creates the doubly near-far problem and weakens throughput fairness.

User cooperation is an effective method to improve the performance of disadvantaged WDs in WPCNs. In the representative cooperative WPCN, a WD with a stronger channel helps relay the information of a weaker WD to the access point so that the system can obtain a more balanced throughput performance [8]. This idea has been further extended to cooperative transmission with distributed virtual antenna arrays [9] and multi-user collaboration with a selected forwarding node [10]. These studies show that relay-assisted transmission can improve fairness and coverage in wireless-powered networks. Nevertheless, cooperation also introduces an additional local information collection process. Before forwarding source information, the relay must first receive the messages from the source WDs. If this process relies on active RF transmission, source WDs must spend additional harvested energy and time before relay forwarding, especially when multiple sources share the same relay.

Backscatter communication provides a low-power way to realize local information exchange. Instead of generating an active RF carrier, a backscatter transmitter conveys data by modulating and reflecting an incident RF signal. Recent surveys have summarized the architecture, circuit implementation, networking protocols, and practical challenges of backscatter communication for battery-free IoT systems [11,12]. In addition, signal detection is a key issue for reliable ambient backscatter communication because the backscattered signal is usually weak and coupled with the strong direct-link component. Novel signal detectors and improved energy-based detection methods have therefore been developed to enhance detection reliability in IoT-oriented backscatter systems [13,14]. Performance analysis for hybrid long-short packet transmission has also shown the potential of backscatter communication under practical packet and reliability requirements [15]. These studies demonstrate the feasibility of low-power backscatter transmission. Unlike conventional ambient backscatter systems that rely on uncontrollable external RF sources, WPCNs provide a dedicated DL WET signal. This signal can be reused as both an energy source and a controllable carrier for passive source-to-relay information collection.

The integration of wireless power transfer (WPT) and backscatter communication has motivated a series of backscatter-assisted wireless-powered transmission designs. Backscatter relay communications powered by wireless energy beamforming were studied in [16], and backscatter-aided cooperative relay communications were further investigated in wireless-powered hybrid radio networks [17]. User cooperation in wireless-powered backscatter communication networks was considered in [18]. More recently, cooperative backscatter-aided passive relaying was studied for wireless-powered D2D networks [19], optimal time allocation was investigated for backscatter-aided relay cooperative transmission in wireless-powered heterogeneous cognitive radio networks [20], and backscatter-assisted wireless-powered MEC with user cooperation was studied in [21]. Multi-backscatter-assisted WPCNs have also been explored for green IoT applications [22]. These works confirm the benefit of RF-signal reuse for low-power transmission and relay assistance. However, they mainly focus on two-user cooperation, relay mode selection, passive relaying, MEC offloading, or multi-tag backscatter transmission, while the energy-recycling role of active UL transmissions among multiple source WDs remains underexplored.

To further clarify the differences from existing studies, Table 1 compares representative backscatter-aided wireless-powered studies in terms of core optimization variables, energy model, fairness objective, and key limitation.

Different from the above works, this paper focuses on the order-dependent energy recycling effect in a multi-source cooperative WPSN. Existing studies mainly exploit backscatter communication to reduce the energy cost of cooperation, while the active UL phase is still commonly treated as an information delivery process. In the considered network, however, the active signal transmitted by one source WD can also be harvested by the source WDs scheduled later. Thus, active UL transmissions act not only as information-bearing signals, but also as recyclable RF energy sources for subsequent transmissions. More importantly, the source transmission order determines which inter-source energy-recycling links are available before each source transmission. Hence, different orders induce different directed energy-recycling structures, reshape the energy-causality constraints, and change the feasible transmit-energy region. From the perspective of throughput fairness among sensor devices, this order-aware mechanism provides an additional degree of freedom to regulate the energy availability of bottleneck source WDs.

**Figure 1:**
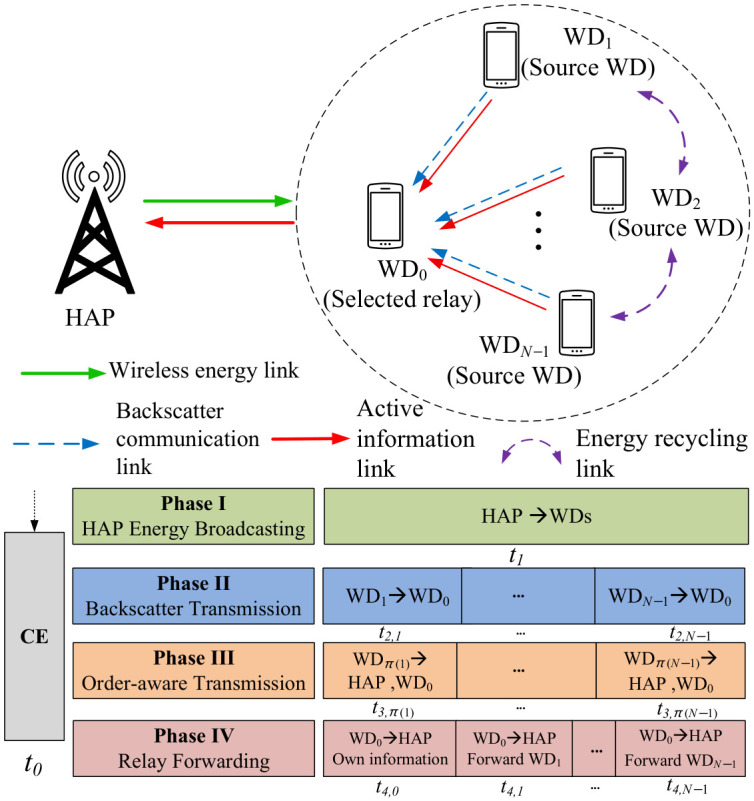
System model and transmission protocol of the proposed scheme.

Motivated by this observation, we propose an order-aware energy recycling scheme for backscatter-assisted cooperative relaying in a single-HAP WPSN. As illustrated in Figure 1, the HAP first broadcasts RF energy to power all WDs. Then, source WDs sequentially backscatter the incident WET signal to deliver their information to the selected relay WD. After that, source WDs actively transmit according to an optimized order, while the later scheduled sources recycle energy from the preceding active UL transmissions. Finally, the relay forwards the decoded source information to the HAP. The proposed protocol jointly exploits passive source-to-relay information collection, active relay-assisted forwarding, and order-aware inter-source energy recycling.

The main contributions of this paper are summarized as follows.

- We develop a backscatter-aided wireless-powered cooperative transmission protocol with order-aware energy recycling. The source WDs reuse the DL WET signal as a controllable carrier for passive source-to-relay information transmission. During the subsequent active UL phase, each scheduled source WD not only transmits information to the relay and the HAP but also acts as a temporary RF energy source for the source WDs scheduled later. Therefore, the source transmission order induces a directed energy-recycling structure among source WDs and determines how recyclable UL energy is redistributed before each source transmission.
- A max-min throughput optimization problem is formulated to improve throughput fairness among sensor devices by jointly designing the source transmission order, time allocation, and transmit-power allocation under energy-causality constraints. Unlike conventional scheduling designs, the source order changes the feasible transmit-energy region because each source WD can only recycle energy from the sources scheduled before it. For a given source transmission order, the continuous resource allocation problem is transformed into a convex form by introducing transmit-energy variables. The optimal order is then obtained by searching over all candidate source transmission orders.
- Numerical results reveal how relay selection, source ordering, and propagation conditions affect the gain of order-aware energy recycling. The proposed scheme achieves higher minimum throughput than the three representative comparison schemes. The performance improvement comes from the joint effect of passive source-to-relay information collection, relay-assisted forwarding, and order-controlled redistribution of recyclable UL energy among source WDs.

The remainder of this paper is organized as follows. Section 2 presents the system model and transmission protocol. Section 3 analyzes the harvested energy and achievable throughput. Section 4 formulates the max-min throughput optimization problem and develops the solution method. Section 5 provides numerical results and performance comparisons. Section 6 concludes this paper.

## 2. System Model

### 2.1. Channel Model

As shown in Figure 1, we consider a backscatter-aided wireless-powered cooperative network consisting of one single-antenna HAP and *N* single-antenna WDs. The HAP has a stable energy supply and broadcasts radio-frequency (RF) energy in the DL, while receiving information in the UL. The WDs rely on the harvested RF energy for active information transmission.

One WD is selected as the relay before each transmission block and is reindexed as ${\mathrm{WD}}_{0}$. The remaining WDs serve as source WDs, indexed by N={1,…,N−1}. The set of all WDs is denoted by $N_{0}=\left\{0\right\}\cup N$. The relay selection follows a predetermined rule before resource allocation and is not optimized in this work. Each source ${\mathrm{WD}}_{i}$, i∈N, delivers its information to the HAP through direct UL transmission and relay-assisted forwarding.

The HAP is equipped with RF communication and wireless energy transfer circuits. Each source WD supports energy harvesting, backscatter transmission, and active RF transmission. The relay has RF communication and energy harvesting circuits, together with a backscatter receiver for decoding the information backscattered by the source WDs. During backscatter transmission, the relay uses separate backscatter-detection and energy-harvesting branches. The associated receiver insertion loss is neglected for analytical tractability.

We adopt a time-division duplexing protocol and assume channel reciprocity for all links. All channels experience quasi-static flat fading, where the channel coefficients remain constant within each transmission block and may vary independently across different blocks. Let $\alpha_{i}$ and $h_{i}={\left|\alpha_{i}\right|}^{2}$, $i\in N_{0}$, denote the channel coefficient and channel power gain between the HAP and ${\mathrm{WD}}_{i}$, respectively. In particular, $\alpha_{0}$ and $h_{0}$ characterize the HAP-to-relay link.

Let $\alpha_{0,i}$ and $h_{0,i}={\left|\alpha_{0,i}\right|}^{2}$, i∈N, and denote the channel coefficient and channel power gain between the relay and source ${\mathrm{WD}}_{i}$. For two different source WDs, $\alpha_{i,j}$ and $h_{i,j}={\left|\alpha_{i,j}\right|}^{2}$, i,j∈N, i≠j, denote the inter-source channel coefficient and channel power gain. These inter-source links allow the source WDs scheduled later to recycle energy from the active UL signals transmitted by the preceding source WDs.

During the channel estimation (CE) phase, the HAP acquires the channel state information (CSI) of the HAP-WD links, relay-source links, and inter-source links, which is used for source ordering and resource allocation.

This perfect-CSI assumption is adopted as a benchmark setting, following common optimization-oriented WPCN studies [4,10]. The required CSI is limited to the selected cooperative transmission block. Nevertheless, the inter-source CSI acquisition introduces additional overhead: with N−1 source WDs, the number of directed inter-source links is (N−1)(N−2), which scales as $O(N^{2})$. Therefore, the cooperative group size should be properly controlled in dense IoT deployments through device grouping, relay-load control, or access control. Imperfect CSI may lead to suboptimal source ordering and inaccurate resource allocation. Therefore, the results under perfect CSI should be interpreted as an upper-bound benchmark for the proposed mechanism, while robust order-aware energy recycling under CSI uncertainty requires an additional error model and robust optimization formulation.

### 2.2. Protocol Description

Figure 1 also illustrates the transmission protocol of the proposed scheme. Each transmission block begins with a CE phase of duration $t_{0}$. The relay is selected before resource allocation and remains unchanged within the transmission block. After the CE phase, the proposed protocol consists of four phases.

In Phase I, the HAP broadcasts an RF energy signal to all WDs for a duration of $t_{1}$. Both the relay and the source WDs harvest energy from the received RF signal and store it for subsequent active information transmission.

In Phase II, the HAP continues broadcasting the RF energy signal, which also serves as the carrier for backscatter communication. The source WDs sequentially transmit their information to the relay through passive backscatter communication. Specifically, source ${\mathrm{WD}}_{i}$, i∈N, is allocated a backscatter duration of $t_{2,i}$. During this interval, ${\mathrm{WD}}_{i}$ reflects part of the incident RF signal to convey its information to the relay and harvests energy from the remaining part. The other source WDs operate in the energy harvesting mode. The relay detects the backscattered information while harvesting energy from the received RF signal through its separate reception branches. The HAP does not decode the backscattered information in this phase.

In Phase III, the source WDs actively transmit according to an optimized order. Let π=[π(1),π(2),…,π(N−1)] denote a permutation of N, where ${\mathrm{WD}}_{\pi (m)}$ is scheduled in the *m*th active transmission slot. During $t_{3,\pi (m)}$, ${\mathrm{WD}}_{\pi (m)}$ broadcasts its information to both the relay and the HAP. Meanwhile, the source WDs scheduled after ${\mathrm{WD}}_{\pi (m)}$ recycle energy from its active UL signal. Therefore, the source transmission order determines not only the active transmission sequence, but also the directed energy-recycling structure among source WDs.

Each source employs rate-compatible coding across Phases II and III. The relay accumulates the information received from the backscatter and active transmission phases before decoding each source message. The HAP stores the directly received information in Phase III for subsequent combination with the corresponding relay transmission.

In Phase IV, the relay first transmits its own information to the HAP during $t_{4,0}$. It then sequentially forwards additional coded information for the source WDs, where $t_{4,i}$, i∈N denotes the forwarding duration for source ${\mathrm{WD}}_{i}$. The HAP combines the information directly received from each source in Phase III with the corresponding information forwarded by the relay in Phase IV.

Accordingly, the overall time allocation satisfies

(1) $$t_{0}+t_{1}+\sum_{i\in N}t_{2,i}+\sum_{i\in N}t_{3,i}+\sum_{i\in N_{0}}t_{4,i}\leq T.$$

For convenience, the transmission block duration is normalized to T=1 in the subsequent analysis.

## 3. Throughput Performance Analysis

### 3.1. Phase I: Wireless Energy Transfer

During Phase I, the HAP broadcasts a normalized RF energy signal $x_{1}\left(t\right)$ with transmit power $P_{1}$ for a duration of $t_{1}$, where $E\left[|x_{1}{\left(t\right)|}^{2}\right]=1.$ The received signal at ${\mathrm{WD}}_{i}$ is

(2) $$y_{i}^{\left(1\right)}\left(t\right)=\alpha_{i}\sqrt{P_{1}}x_{1}\left(t\right)+n_{i}^{\left(1\right)}\left(t\right),\;i\in N_{0},$$

where $n_{i}^{\left(1\right)}\left(t\right)$ is the additive white Gaussian noise with power $N_{0}$.

Ignoring the negligible energy harvested from receiver noise, the energy harvested by ${\mathrm{WD}}_{i}$ during Phase I is

(3) $$E_{i}^{\left(1\right)}=\eta P_{1}h_{i}t_{1},\;i\in N_{0},$$

where 0<η<1 is the RF-to-DC energy conversion efficiency.

### 3.2. Phase II: Source-to-Relay Backscatter Transmission

During Phase II, the HAP continues broadcasting an RF energy signal with power $P_{1}$. The source WDs sequentially backscatter the incident signal to transmit their information to the relay. Source ${\mathrm{WD}}_{i}$, i∈N, is allocated a backscatter duration of $t_{2,i}$.

Let $x_{2}\left(t\right)$ denote the normalized RF signal transmitted by the HAP, with  $E\left[|x_{2}{\left(t\right)|}^{2}\right]=1.$ Let $o_{i}\left(t\right)$ denote the normalized information-bearing backscatter symbol of ${\mathrm{WD}}_{i}$, where $E\left[o_{i}\left(t\right)\right]=0$ and $E\left[|o_{i}{\left(t\right)|}^{2}\right]=1$. The reflection coefficient of ${\mathrm{WD}}_{i}$ is denoted by $\mu_{i}$, with $0\leq \mu_{i}\leq 1$. The signal backscattered by ${\mathrm{WD}}_{i}$ is

(4) $$s_{i}^{\left(2\right)}\left(t\right)=\sqrt{\mu_{i}}\alpha_{i}\sqrt{P_{1}}x_{2}\left(t\right)o_{i}\left(t\right),\;i\in N.$$

Accordingly, the received signal at the relay is

(5) $$\begin{array}{c} y_{0,i}^{\left(2\right)}\left(t\right)=\sqrt{\mu_{i}}\alpha_{0,i}\alpha_{i}\sqrt{P_{1}}x_{2}\left(t\right)o_{i}\left(t\right)+\alpha_{0}\sqrt{P_{1}}x_{2}\left(t\right)+n_{0,i}^{\left(2\right)}\left(t\right),\;i\in N, \end{array}$$

where $n_{0,i}^{\left(2\right)}\left(t\right)$ is the additive white Gaussian noise at the relay with power $N_{0}$. The relay splits the received RF signal into the detection and energy-harvesting branches. In the detection branch, the direct HAP component is suppressed before decoding the backscattered information.

During its own backscatter interval, ${\mathrm{WD}}_{i}$ reflects a fraction $\mu_{i}$ of the incident RF power and harvests the remaining fraction. During the other backscatter intervals, it operates in the energy harvesting mode. The weak energy carried by backscattered signals from other source WDs is neglected due to the double-path attenuation. Hence, the energy harvested by ${\mathrm{WD}}_{i}$ during Phase II is

(6) $$\begin{array}{c} E_{i}^{\left(2\right)}=\eta P_{1}h_{i}\left(\sum_{\begin{array}{c} j\in N,j\neq i \end{array}}t_{2,j}+\left(1-\mu_{i}\right)t_{2,i}\right),\;i\in N. \end{array}$$

Together with Phase I, the total energy harvested by source ${\mathrm{WD}}_{i}$ from the HAP before active transmission is

(7) $$\begin{array}{c} E_{i}^{H}=E_{i}^{\left(1\right)}+E_{i}^{\left(2\right)}=\eta P_{1}h_{i}\left(t_{1}+\sum_{j\in N}t_{2,j}-\mu_{i}t_{2,i}\right),\;i\in N. \end{array}$$

The approximation error introduced by neglecting cross-source backscatter harvested energy can be measured by the ratio between the neglected backscatter energy and the direct HAP-to-source harvested energy. During the backscatter interval of ${\mathrm{WD}}_{j}$, j≠i, this relative error for ${\mathrm{WD}}_{i}$ can be expressed as $\epsilon_{j\to i}^{\mathrm{BS}}=\mu_{j}h_{j}h_{j,i}/h_{i}$. Therefore, this simplification is valid when $\mu_{j}h_{j}h_{j,i}\ll h_{i}$, which generally holds for far-field low-power backscatter links due to cascaded propagation loss and reflection loss [16,17].

During the backscatter interval of ${\mathrm{WD}}_{i}$, the relay harvests energy from both the direct HAP signal and the reflected signal. Since $E[o_{i}\left(t\right)]=0$, the cross term vanishes after averaging. The energy harvested by the relay during $t_{2,i}$ is therefore

(8) $$E_{0}^{\left(2,i\right)}=\eta P_{1}\left(h_{0}+\mu_{i}h_{0,i}h_{i}\right)t_{2,i},\;i\in N.$$

Thus, the total energy harvested by the relay during Phase II is

(9) $$E_{0}^{\left(2\right)}=\sum_{i\in N}E_{0}^{\left(2,i\right)}.$$

Considering the effects of the selected reflection coefficient, backscatter circuit, channel quality, detector performance, and coding overhead, the successfully delivered information from ${\mathrm{WD}}_{i}$ to the relay is modeled as

(10) $$R_{i,0}^{\left(2\right)}\left(t\right)=C_{i}R_{b}t_{2,i},\;i\in N,$$

where $R_{b}$ is the nominal backscatter transmission rate and $0\leq C_{i}\leq 1$ is the effective successful-information ratio of the backscatter link.

The practical hardware impairments are reflected by the effective protocol-level parameters in (10). RF circuit loss and rectifier loss reduce the effective energy conversion efficiency η. Backscatter modulation power consumption decreases the net harvested energy available for later active transmission, and can be incorporated by subtracting a circuit-energy term such as $P_{c,i}^{\mathrm{BS}}t_{2,i}$ from the harvested energy of ${\mathrm{WD}}_{i}$. Phase noise, synchronization mismatch, imperfect carrier suppression, and detector errors reduce backscatter decoding reliability, which can be captured by a smaller successful-information ratio $C_{i}$ or a lower effective backscatter rate $R_{b}$. These impairments may reduce the absolute throughput, but they do not change the proposed order-aware energy-recycling mechanism.

### 3.3. Phase III: Order-Aware Active Transmission and Energy Recycling

During Phase III, the source WDs actively transmit according to the order π. Source ${\mathrm{WD}}_{\pi (m)}$ is scheduled in the *m*th active transmission slot with duration $t_{3,\pi (m)}$, where m∈N.

Before its active transmission, ${\mathrm{WD}}_{\pi (m)}$ can recycle energy from the UL signals transmitted by the preceding sources ${\mathrm{WD}}_{\pi (\ell )}$, ℓ=1,…,m−1. The recycled energy is given by

(11) $$E_{\pi (m)}^{\mathrm{rec}}=\eta \sum_{\ell =1}^{m-1}t_{3,\pi (\ell )}P_{3,\pi (\ell )}h_{\pi (\ell ),\pi (m)},\;m\in N,$$

where the summation is zero for m=1.

Thus, the active transmission of ${\mathrm{WD}}_{\pi (m)}$ must satisfy

(12) $$\begin{array}{cc} t_{3,\pi (m)}P_{3,\pi (m)}\leq & \eta P_{1}h_{\pi (m)}\left(t_{1}+\sum_{j\in N}t_{2,j}-\mu_{\pi (m)}t_{2,\pi (m)}\right)\\ \; & +\eta \sum_{\ell =1}^{m-1}t_{3,\pi (\ell )}P_{3,\pi (\ell )}h_{\pi (\ell ),\pi (m)},\;m\in N. \end{array}$$

Constraint (12) shows that the source order determines which inter-source energy-recycling links are available before each active transmission. Hence, different orders lead to different energy-causality constraints and feasible source transmit-energy regions.

Let $x_{3,i}\left(t\right)$ denote the normalized information signal transmitted by source ${\mathrm{WD}}_{i}$, with 

$$E\left[|x_{3,i}{\left(t\right)|}^{2}\right]=1,\;i\in N.$$

During the active transmission of ${\mathrm{WD}}_{i}$, the relay and the HAP receive

(13) $$y_{i,0}^{\left(3\right)}\left(t\right)=\alpha_{0,i}\sqrt{P_{3,i}}x_{3,i}\left(t\right)+n_{0,i}^{\left(3\right)}\left(t\right),\;i\in N,$$

and

(14) $$y_{i,H}^{\left(3\right)}\left(t\right)=\alpha_{i}\sqrt{P_{3,i}}x_{3,i}\left(t\right)+n_{H,i}^{\left(3\right)}\left(t\right),\;i\in N,$$

respectively, where $n_{0,i}^{\left(3\right)}\left(t\right)$ and $n_{H,i}^{\left(3\right)}\left(t\right)$ are independent additive white Gaussian noises with power $N_{0}$.

The achievable active transmission rates from ${\mathrm{WD}}_{i}$ to the relay and to the HAP are respectively given by

(15) $$R_{i,0}^{\left(3\right)}\left(t,P_{3}\right)=t_{3,i}\log_{2}\left(1+\frac{P_{3,i}h_{0,i}}{N_{0}}\right),\;i\in N,$$

and

(16) $$R_{i,H}^{\left(3\right)}\left(t,P_{3}\right)=t_{3,i}\log_{2}\left(1+\frac{P_{3,i}h_{i}}{N_{0}}\right),\;i\in N.$$

Therefore, Phase III supports both active information transmission and order-aware energy recycling among source WDs.

### 3.4. Phase IV: Relay Forwarding

During Phase IV, the relay first transmits its own information to the HAP and then forwards additional coded information for the source WDs. The relay uses duration $t_{4,i}$ and transmit power $P_{4,i}$ for the information associated with ${\mathrm{WD}}_{i}$, where $i\in N_{0}$. Here, i=0 corresponds to the relay’s own information, while i∈N corresponds to source information forwarding.

Let $x_{4,i}\left(t\right)$ denote the normalized signal transmitted by the relay for ${\mathrm{WD}}_{i}$, with  $E\left[|x_{4,i}{\left(t\right)|}^{2}\right]=1,i\in N_{0}$. The received signal at the HAP is

(17) $$y_{i,H}^{\left(4\right)}\left(t\right)=\alpha_{0}\sqrt{P_{4,i}}x_{4,i}\left(t\right)+n_{i,H}^{\left(4\right)}\left(t\right),\;i\in N_{0},$$

where $n_{i,H}^{\left(4\right)}\left(t\right)$ is the additive white Gaussian noise at the HAP with power $N_{0}$.

The relay transmission energy is constrained by the energy harvested in Phases I and II:

(18) $$\sum_{i\in N_{0}}t_{4,i}P_{4,i}\leq E_{0}^{\left(1\right)}+E_{0}^{\left(2\right)}.$$

The achievable rate for forwarding the information of source ${\mathrm{WD}}_{i}$ is

(19) $$R_{i,H}^{\left(4\right)}\left(t,P_{4}\right)=t_{4,i}\log_{2}\left(1+\frac{P_{4,i}h_{0}}{N_{0}}\right),\;i\in N.$$

Following the information-accumulation mechanism, the relay combines the backscatter information received in Phase II and the active information received in Phase III. The accumulated information at the relay is

(20) $$R_{i,0}^{\mathrm{acc}}=R_{i,0}^{\left(2\right)}\left(t\right)+R_{i,0}^{\left(3\right)}\left(t,P_{3}\right),\;i\in N.$$

The HAP combines the direct information received in Phase III and the relay-forwarded information received in Phase IV. The accumulated information at the HAP is

(21) $$R_{i,H}^{\mathrm{acc}}=R_{i,H}^{\left(3\right)}\left(t,P_{3}\right)+R_{i,H}^{\left(4\right)}\left(t,P_{4}\right),\;i\in N.$$

With decode-and-forward relaying, the end-to-end throughput of source ${\mathrm{WD}}_{i}$ is

(22) $$R_{i}\left(t,P_{3},P_{4}\right)=\min\left\{R_{i,0}^{\mathrm{acc}},R_{i,H}^{\mathrm{acc}}\right\},\;i\in N.$$

The achievable throughput of the relay itself is

(23) $$R_{0}\left(t,P_{4}\right)=t_{4,0}\log_{2}\left(1+\frac{P_{4,0}h_{0}}{N_{0}}\right).$$

Although π does not explicitly appear in the rate expressions, it determines the energy-causality constraint in (12) and thereby affects the feasible source transmit powers and achievable throughputs.

## 4. Max-Min Throughput Optimization

### 4.1. Problem Formulation

We jointly optimize the source transmission order, time allocation, and transmit-power allocation to maximize the minimum achievable throughput among all WDs. The time allocation vector is defined as $t=\left[t_{1},{\left\{t_{2,i}\right\}}_{i\in N},{\left\{t_{3,i}\right\}}_{i\in N},{\left\{t_{4,i}\right\}}_{i\in N_{0}}\right],$ and the source and relay transmit-power vectors are defined as $P_{3}={\left[P_{3,i}\right]}_{i\in N},P_{4}={\left[P_{4,i}\right]}_{i\in N_{0}}.$ Let P denote the set of all permutations of N. The max-min throughput optimization problem is formulated as

(24a) $$\begin{array}{cc} \left(P1\right):\;\max_{\begin{array}{c} \pi ,t,P_{3},P_{4} \end{array}}\; & \min_{i\in N_{0}}R_{i}\left(t,P_{3},P_{4}\right) \end{array}$$

(24b) $$\begin{array}{cc} s.t.\; & t_{0}+t_{1}+\sum_{i\in N}t_{2,i}+\sum_{i\in N}t_{3,i}+\sum_{i\in N_{0}}t_{4,i}\leq 1,\\ \; & t_{3,\pi (m)}P_{3,\pi (m)}\leq \eta P_{1}h_{\pi (m)}\left(t_{1}+\sum_{j\in N}t_{2,j}-\mu_{\pi (m)}t_{2,\pi (m)}\right) \end{array}$$

(24c) $$\begin{array}{cc} \; & \;+\eta \sum_{\ell =1}^{m-1}t_{3,\pi (\ell )}P_{3,\pi (\ell )}h_{\pi (\ell ),\pi (m)},\;m\in N, \end{array}$$

(24d) $$\begin{array}{cc} \; & \sum_{i\in N_{0}}t_{4,i}P_{4,i}\leq E_{0}^{\left(1\right)}+E_{0}^{\left(2\right)}, \end{array}$$

(24e) $$\begin{array}{cc} \; & \pi \in P, \end{array}$$

(24f) $$\begin{array}{cc} \; & t_{1}\geq 0, \end{array}$$

(24g) $$\begin{array}{cc} \; & t_{2,i}\geq 0,t_{3,i}\geq 0,P_{3,i}\geq 0,i\in N, \end{array}$$

(24h) $$\begin{array}{cc} \; & t_{4,i}\geq 0,P_{4,i}\geq 0,i\in N_{0}. \end{array}$$

Constraint (24c) describes the order-dependent energy causality of the source WDs. It shows that the feasible transmit energy of ${\mathrm{WD}}_{\pi (m)}$ is determined by its harvested energy from the HAP and the recycled energy from the preceding active UL transmissions.

Constraint (24d) limits the relay transmit energy by its harvested energy in Phases I and II. Substituting (3) and (8) gives

(25) $$\begin{array}{cc} \sum_{i\in N_{0}}t_{4,i}P_{4,i}\leq & \eta P_{1}h_{0}t_{1}+\eta P_{1}\sum_{i\in N}\left(h_{0}+\mu_{i}h_{0,i}h_{i}\right)t_{2,i}. \end{array}$$

Problem (P1) is a mixed discrete-continuous optimization problem. The source order π is discrete, while the time and power variables are coupled in the throughput expressions and energy-causality constraints. We solve it by first optimizing the continuous resource allocation for a given source order and then searching for the optimal order.

### 4.2. Resource Allocation for a Given Transmission Order

For a given source transmission order π, we introduce the source and relay transmission-energy variables as $\tau_{3,i}=t_{3,i}P_{3,i},i\in N,\tau_{4,i}=t_{4,i}P_{4,i},i\in N_{0}$. Let $\tau_{3}={\left[\tau_{3,i}\right]}_{i\in N},\tau_{4}={\left[\tau_{4,i}\right]}_{i\in N_{0}}.$ With these variables, the order-dependent source energy constraints become

(26) $$\begin{array}{cc} \tau_{3,\pi (m)}\leq & \eta P_{1}h_{\pi (m)}\left(t_{1}+\sum_{j\in N}t_{2,j}-\mu_{\pi (m)}t_{2,\pi (m)}\right)\\ \; & +\eta \sum_{\ell =1}^{m-1}\tau_{3,\pi (\ell )}h_{\pi (\ell ),\pi (m)},\;m\in N, \end{array}$$

where the summation is zero for m=1. Similarly, the relay energy constraint is rewritten as

(27) $$\sum_{i\in N_{0}}\tau_{4,i}\leq \eta P_{1}h_{0}t_{1}+\eta P_{1}\sum_{i\in N}\left(h_{0}+\mu_{i}h_{0,i}h_{i}\right)t_{2,i}.$$

Define $\rho_{1,i}=\frac{h_{0,i}}{N_{0}},\rho_{2,i}=\frac{h_{i}}{N_{0}},i\in N,\rho_{3}=\frac{h_{0}}{N_{0}}$. The active source-to-relay, source-to-HAP, relay-forwarding, and relay-own rates can be expressed as

(28) $${\tilde{R}}_{i,0}^{\left(3\right)}\left(t_{3,i},\tau_{3,i}\right)=t_{3,i}\log_{2}\left(1+\rho_{1,i}\frac{\tau_{3,i}}{t_{3,i}}\right),\;i\in N,$$

(29) $${\tilde{R}}_{i,H}^{\left(3\right)}\left(t_{3,i},\tau_{3,i}\right)=t_{3,i}\log_{2}\left(1+\rho_{2,i}\frac{\tau_{3,i}}{t_{3,i}}\right),\;i\in N,$$

(30) $${\tilde{R}}_{i,H}^{\left(4\right)}\left(t_{4,i},\tau_{4,i}\right)=t_{4,i}\log_{2}\left(1+\rho_{3}\frac{\tau_{4,i}}{t_{4,i}}\right),\;i\in N,$$

(31) $${\tilde{R}}_{0}\left(t_{4,0},\tau_{4,0}\right)=t_{4,0}\log_{2}\left(1+\rho_{3}\frac{\tau_{4,0}}{t_{4,0}}\right).$$

The above perspective functions are interpreted in the closed perspective sense: they are defined as zero when the corresponding time and transmission-energy variables are both zero, while the case with zero duration and positive transmission energy is excluded from the effective domain.

For the given order π, we introduce an auxiliary variable $\bar{R}$ for the minimum throughput. Problem (P1) is then reformulated as

(32a) $$\left(P2\right)\left(\pi \right):\;\max_{\begin{array}{c} \bar{R},t,\tau_{3},\tau_{4} \end{array}}\;\bar{R}$$

(32b) $$\begin{array}{cc} s.t.\;\; & t_{0}+t_{1}+\sum_{i\in N}t_{2,i}+\sum_{i\in N}t_{3,i}+\sum_{i\in N_{0}}t_{4,i}\leq 1,\\ \; & \tau_{3,\pi (m)}\leq \eta P_{1}h_{\pi (m)}\left(t_{1}+\sum_{j\in N}t_{2,j}-\mu_{\pi (m)}t_{2,\pi (m)}\right) \end{array}$$

(32c) $$\;+\eta \sum_{\ell =1}^{m-1}\tau_{3,\pi (\ell )}h_{\pi (\ell ),\pi (m)},\;m\in N,$$

(32d) $$\sum_{i\in N_{0}}\tau_{4,i}\leq \eta P_{1}h_{0}t_{1}+\eta P_{1}\sum_{i\in N}\left(h_{0}+\mu_{i}h_{0,i}h_{i}\right)t_{2,i},$$

(32e) $$\bar{R}\leq C_{i}R_{b}t_{2,i}+{\tilde{R}}_{i,0}^{\left(3\right)}\left(t_{3,i},\tau_{3,i}\right),\;i\in N,$$

(32f) $$\bar{R}\leq {\tilde{R}}_{i,H}^{\left(3\right)}\left(t_{3,i},\tau_{3,i}\right)+{\tilde{R}}_{i,H}^{\left(4\right)}\left(t_{4,i},\tau_{4,i}\right),\;i\in N,$$

(32g) $$\bar{R}\leq {\tilde{R}}_{0}\left(t_{4,0},\tau_{4,0}\right),$$

(32h) $$\bar{R}\geq 0,\;t_{1}\geq 0,$$

(32i) $$t_{2,i}\geq 0,\;t_{3,i}\geq 0,\;\tau_{3,i}\geq 0,\;i\in N,$$

(32j) $$t_{4,i}\geq 0,\;\tau_{4,i}\geq 0,\;i\in N_{0}.$$

The rate functions in (28)–(31) are perspectives of concave logarithmic functions and are jointly concave in the corresponding time and energy variables. For a given order π, the energy constraints in (32c) and (32d) are affine, and the throughput constraints in (32e)–(32g) define hypographs of concave functions. Therefore, (P2)(π) is a convex optimization problem and can be solved by standard interior-point methods.

After solving (P2)(π), the optimal source and relay transmit powers are recovered as

(33) $$P_{3,i}^{*}=\left\{\begin{array}{cc} \frac{\tau_{3,i}^{*}}{t_{3,i}^{*}},\; & t_{3,i}^{*}>0,\\ 0,\; & t_{3,i}^{*}=0, \end{array}\right.\;i\in N,$$

and

(34) $$P_{4,i}^{*}=\left\{\begin{array}{cc} \frac{\tau_{4,i}^{*}}{t_{4,i}^{*}},\; & t_{4,i}^{*}>0,\\ 0,\; & t_{4,i}^{*}=0, \end{array}\right.\;i\in N_{0}.$$

An optimal solution can always be chosen with zero transmission energy for any zero-duration transmission interval.

### 4.3. Transmission Order Optimization

Let ${\bar{R}}^{*}\left(\pi \right)$ denote the optimal objective value of (P2)(π). The optimal source transmission order is obtained as

(35) $$\pi^{*}=\arg\max_{\pi \in P}{\bar{R}}^{*}\left(\pi \right),$$

where P contains all permutations of N.

The exact solution is obtained by enumerating all (N−1)! candidate orders. For each π∈P, the convex problem (P2)(π) is solved globally. The order with the largest ${\bar{R}}^{*}\left(\pi \right)$ is selected as $\pi^{*}$. Hence, the exhaustive-search procedure obtains the global optimum of (P1). The overall procedure is summarized in Algorithm 1.

The proposed exact method terminates after a finite search over (N−1)! source orders, where each (P2)(π) is solved as a convex program. In the simulations, the convex subproblems were implemented in MATLAB R2024b using CVX 2.2 with the default solver SDPT3 4.0. The default CVX precision was used, whose “Solved” criterion corresponds to a precision no larger than $1.49\times 10^{-8}$. The maximum solver iteration number was not manually specified, and no manual initialization of the optimization variables was required.  
**Algorithm 1** Joint transmission order and resource allocation**Input:** Channel gains, system parameters, and the source WD set N.**Output:** Optimal source order $\pi^{*}$, time allocation $t^{*}$, transmit-power allocation $P_{3}^{*}$ and$P_{4}^{*}$, and maximum common throughput ${\bar{R}}_{\max}$.  1:Generate the permutation set P of N.  2:Set ${\bar{R}}_{\max}=-\infty$.  3:**for** each π∈P **do**  4:      Solve (P2)(π), and obtain ${\bar{R}}^{*}\left(\pi \right)$, $t^{*}\left(\pi \right)$, $\tau_{3}^{*}\left(\pi \right)$, and $\tau_{4}^{*}\left(\pi \right)$.  5:      **if** ${\bar{R}}^{*}\left(\pi \right)>{\bar{R}}_{\max}$ **then**  6:            Set ${\bar{R}}_{\max}={\bar{R}}^{*}\left(\pi \right)$ and $\pi^{*}=\pi$.  7:            Set $t^{*}=t^{*}\left(\pi \right)$, $\tau_{3}^{*}=\tau_{3}^{*}\left(\pi \right)$, and $\tau_{4}^{*}=\tau_{4}^{*}\left(\pi \right)$.  8:      **end if**  9:**end for**10:Recover $P_{3}^{*}$ and $P_{4}^{*}$ using (33) and (34).11:**return** $\pi^{*}$, $t^{*}$, $P_{3}^{*}$, $P_{4}^{*}$, and ${\bar{R}}_{\max}$.

Let $T_{P2}$ denote the complexity of solving one instance of (P2)(π). Since one convex problem is solved for each candidate order, the overall complexity is $O\left(\left(N-1\right)!T_{P2}\right)$. Therefore, the exact method is suitable for a moderate number of source WDs and can serve as a globally optimal benchmark for lower-complexity ordering methods. Although the exhaustive search provides the optimal source order for the selected cooperative group, its complexity increases factorially with the number of source WDs. Therefore, it is mainly used as an optimal block-level benchmark in this paper. For dense IoT deployments, scalable implementations may require device grouping, relay-load control, access control, or candidate-order pruning to keep the cooperative block size and ordering complexity manageable. In candidate-order pruning, only a limited number of structured candidate orders are evaluated instead of all (N−1)! possible orders, which provides a tunable performance–computation trade-off. A systematic design and validation of such low-complexity ordering methods will be investigated in future work.

## 5. Simulation Results

In this section, numerical results are presented to evaluate the proposed order-aware energy recycling scheme. We consider a single-HAP WPSN, where the HAP is located at the origin and N=5 WDs, including one selected relay WD and four source WDs, are randomly deployed in a circular region centered at (d,0) with radius *r*. Before each transmission block, one WD is selected as the relay, and the remaining WDs act as source WDs. All wireless links experience independent Rayleigh fading with distance-dependent path loss. Specifically, the channel coefficient of a generic link with distance $d_{\ell }$ is generated as $\alpha_{\ell }∼\mathrm{CN}\left(0,\sigma_{\ell }^{2}\right),\sigma_{\ell }^{2}=G_{A}{\left(\frac{3\times 10^{8}}{4\pi d_{\ell }f_{c}}\right)}^{\lambda }$. This model is applied to the HAP-to-WD links, relay-to-source links, and inter-source links with their corresponding link distances. Unless otherwise specified, the HAP transmit power is $P_{1}=2$ W, the energy conversion efficiency is η=0.6, the noise power is $N_{0}=10^{-10}$ W, the carrier frequency is $f_{c}=915$ MHz, the path-loss exponent is λ=2.5, the antenna power gain is $G_{A}=3$, the channel estimation time is $t_{0}=0.05$, and the user-region radius is r=3 m. The backscatter reflection coefficient and the effective successful-information ratio are set to $\mu_{i}=0.8$ and $C_{i}=0.8$, respectively, for all source WDs. The nominal backscatter transmission rate is $R_{b}=10$ bps/Hz. Each data point is averaged over 50 independent random WD deployments and small-scale fading realizations. For fair comparison, all compared schemes use the same deployment and fading realizations under each parameter setting.

We first examine the relay-selection rule used before resource allocation. Three rules are considered: nearest-to-HAP relay selection, nearest-to-center relay selection, and random relay selection. In the nearest-to-HAP rule, the WD closest to the HAP is selected as the relay; in the nearest-to-center rule, the WD closest to the center of the user region is selected; and in the random rule, the relay is randomly selected from all WDs. After the relay is selected, the source transmission order and continuous resource allocation are optimized for the corresponding network realization.

**Figure 2:**
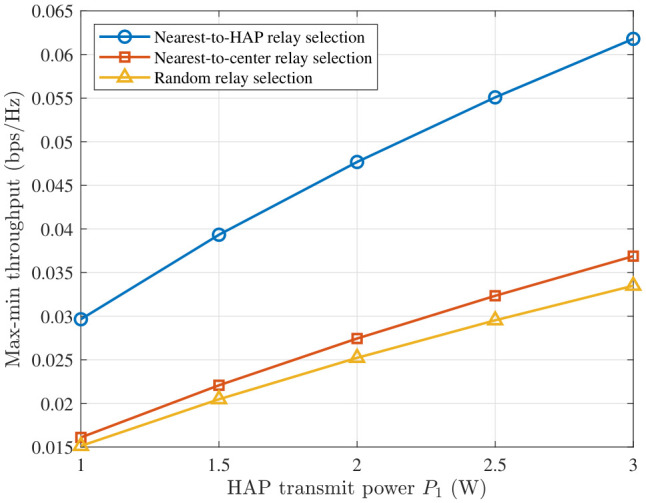
Max-min throughput versus the HAP transmit power $P_{1}$ under different relay-selection rules.

As $P_{1}$ increases in Figure 2, all relay-selection rules achieve higher max-min throughput because the harvested energy at both the source WDs and the relay increases. The nearest-to-HAP rule consistently performs best, indicating that the relay-HAP link is more critical than the average relay-source proximity in the considered protocol. The relay must harvest energy, decode source information, forward source messages, and transmit its own data. A relay close to the HAP can therefore avoid becoming both an energy bottleneck and a forwarding bottleneck. The nearest-to-center rule improves local connectivity to the source WDs, but this gain cannot compensate for a weak relay-HAP link. Random relay selection provides no control over either bottleneck and thus gives the lowest throughput.

**Figure 3:**
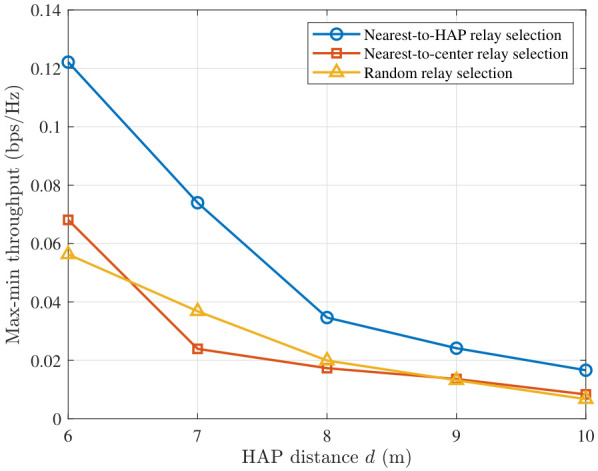
Max-min throughput versus the HAP distance *d* under different relay-selection rules.

The same conclusion is reinforced by Figure 3. Increasing *d* weakens the HAP-to-WD links, so the WDs harvest less energy and require more transmit energy for UL delivery. This double impact explains the sharp throughput degradation. The nearest-to-HAP rule remains the most robust because it preserves the strongest possible relay-HAP path under each deployment. When *d* becomes large, the performance gap among the three rules narrows since severe path loss makes all WDs energy-limited. Nevertheless, the nearest-to-HAP rule still provides the highest throughput and is therefore used as the default relay-selection rule in the following simulations.

**Figure 4:**
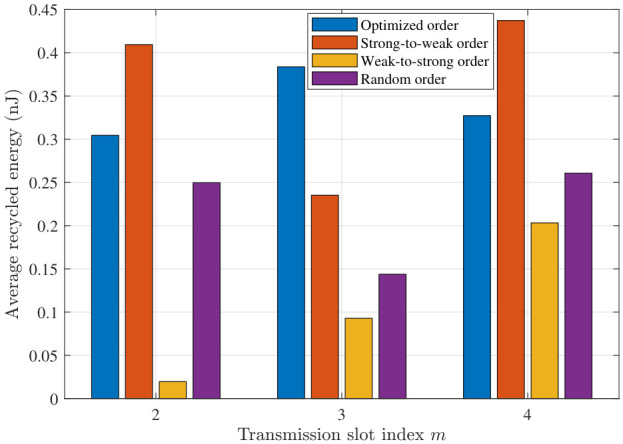
Average recycled energy versus the transmission slot index *m* under different source transmission orders.

Figure 4 illustrates how the source transmission order changes the inter-source energy-recycling pattern. The first scheduled source has no preceding active transmission to harvest from, so the comparison starts from later slots. The weak-to-strong order provides limited recycled energy because weak sources are scheduled early and cannot generate strong RF signals for later sources. The random order produces an unstable energy distribution because it ignores both channel strength and energy demand. The strong-to-weak order can generate a large amount of recycled energy in some slots since strong sources scheduled earlier may transmit with higher energy. However, the optimized order does not simply maximize the recycled energy in every slot. A large amount of recycled energy is useful only when it reaches the sources that limit the minimum throughput and when those sources can convert the extra energy into effective relay/HAP transmission. Therefore, the optimized order balances two coupled roles of each source WD: an earlier source may act as an RF energy contributor, while a later source may act as an energy recycler before its own transmission. This confirms that source ordering reshapes the energy-causality structure rather than serving as a simple scheduling sequence.

The proposed scheme is compared with three schemes. The proposed scheme without OA-ER, where OA-ER denotes order-aware energy recycling, retains WET, backscatter-assisted source-to-relay information collection, and relay forwarding but removes the inter-source energy recycling term in the active UL phase. This scheme is used to isolate the contribution of order-aware energy recycling. Cooperation without backscatter performs source-to-relay information collection through active RF transmission. It retains relay-assisted forwarding, but the source WDs consume additional harvested energy and time before relay forwarding. Direct transmission removes relay-assisted cooperation, and each WD directly transmits to the HAP after energy harvesting. Each scheme optimizes its own time and transmit-power allocation under the corresponding protocol constraints.

**Figure 5:**
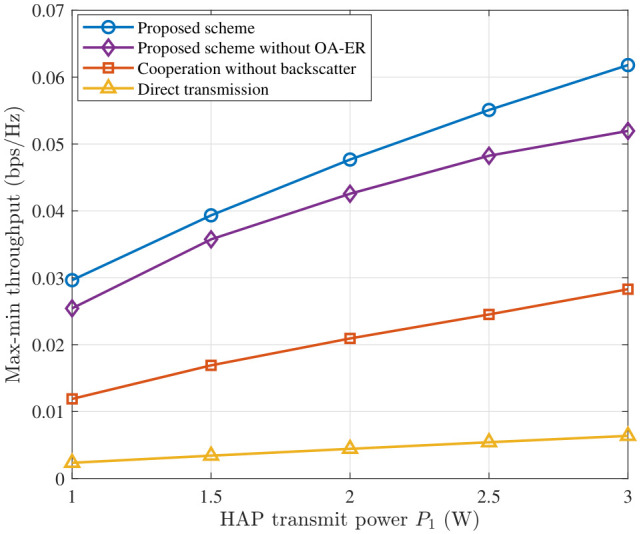
Max-min throughput versus the HAP transmit power $P_{1}$ for different schemes.

The curves in Figure 5 reveal how each scheme converts additional wireless energy into fairness improvement. As $P_{1}$ increases, the feasible transmit-energy region of all WDs expands. The proposed scheme obtains the largest gain because the extra harvested energy supports ordered active source transmission, relay forwarding, and inter-source energy recycling. Compared with the proposed scheme without OA-ER, the proposed scheme further allows later scheduled source WDs to recycle energy from preceding active UL transmissions, thereby enlarging the feasible transmit-energy region of bottleneck WDs. This explains the performance gap between the two schemes and confirms the contribution of order-aware energy recycling. In cooperation without backscatter, part of the harvested energy is consumed by active source-to-relay transmission, which reduces the net energy available for later cooperative phases. Direct transmission also benefits from higher harvested energy, but the additional energy only strengthens direct source-to-HAP links and cannot create relay-assisted information diversity or inter-source energy recycling. This explains why the proposed scheme has a steeper throughput growth.

**Figure 6:**
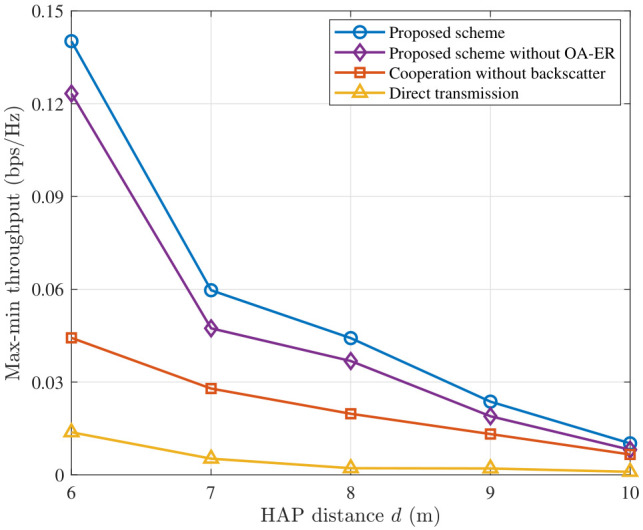
Max-min throughput versus the HAP distance *d* for different schemes.

The throughput degradation with the HAP distance is shown in Figure 6. A larger *d* simultaneously weakens DL energy harvesting and UL information delivery, so all schemes suffer from the doubly near-far effect. The proposed scheme achieves the highest max-min throughput over the entire distance range because it reduces the energy cost of local cooperation and preserves more harvested energy for active UL transmission, relay forwarding, and inter-source energy recycling. The proposed scheme without OA-ER follows a similar cooperative transmission structure, but it cannot use preceding active UL signals as additional RF energy sources for later scheduled WDs. As a result, its feasible transmit-energy region is smaller, especially for bottleneck WDs under weakened HAP-related links. Cooperation without backscatter still benefits from relaying, but its active source-to-relay collection consumes energy before the cooperative gain can be fully realized. Direct transmission has the simplest protocol, but it lacks relay-assisted forwarding, source-to-relay information accumulation, and inter-source energy recycling. Hence, its performance is limited by the weakest direct HAP link.

**Figure 7:**
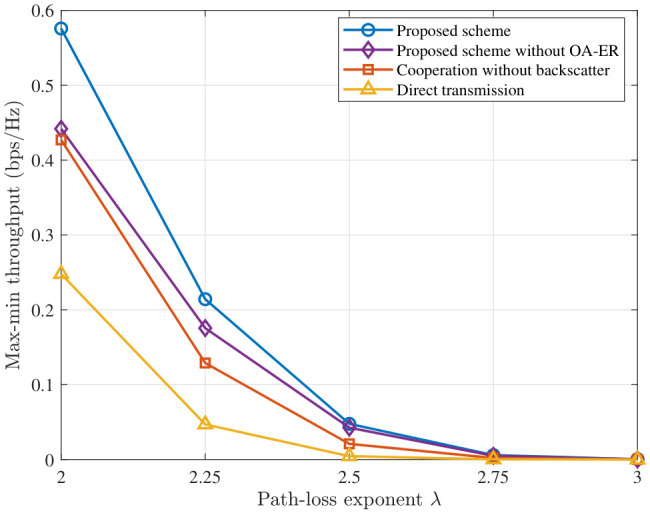
Max-min throughput versus the path-loss exponent λ for different schemes.

A different aspect is captured in Figure 7, where the path-loss exponent λ varies. Increasing λ suppresses all RF links, including the WET links, backscatter links, active source-to-relay links, inter-source energy-recycling links, and relay-to-HAP forwarding link. Therefore, the max-min throughput decreases for all schemes. The proposed scheme still performs best because passive backscatter reduces the local cooperation cost and OA-ER exploits the remaining inter-source RF energy. The curve of the proposed scheme without OA-ER further confirms the role of inter-source energy recycling: once the recycling term is removed, the system loses the additional energy supply created by preceding active UL transmissions. As λ becomes larger, this gap gradually narrows because the recyclable inter-source energy is also weakened by severe path loss. The other two schemes are more sensitive to propagation loss for different reasons. Cooperation without backscatter requires active source-to-relay information collection before relay forwarding, and this additional active exchange becomes costly when RF links are weak. Direct transmission avoids cooperation overhead, but it relies entirely on direct HAP links and therefore cannot benefit from relay-assisted diversity or recycled UL energy. This result shows that OA-ER is most effective when the inter-source and relay-related links are sufficiently strong to support both energy redistribution and cooperative information delivery.

Overall, the results verify that the proposed scheme improves max-min throughput by jointly exploiting passive source-to-relay collection, relay-assisted forwarding, and order-aware energy recycling. The comparison with the proposed scheme without OA-ER confirms that inter-source energy recycling provides additional transmit energy for later scheduled WDs and helps support throughput-limited users. Compared with cooperation without backscatter and direct transmission, the proposed scheme achieves a better balance among local-collection overhead, energy redistribution, and cooperative information delivery.

## 6. Conclusions

This paper investigated order-aware energy recycling for backscatter-aided wireless-powered cooperative communications. The key observation is that active UL transmissions in a multi-source WPSN can serve not only as information-bearing signals but also as order-dependent RF energy sources for later scheduled source WDs. Based on this observation, the source transmission order was jointly designed with time and transmit-power allocation to improve throughput fairness among energy-constrained WDs. For a given order, the continuous resource allocation problem was transformed into a convex problem through transmit-energy variables, and the optimal order was obtained by exhaustive search. Numerical results showed that the proposed scheme improves the minimum throughput over the proposed scheme without OA-ER, cooperation without backscatter, and direct transmission. The ablation comparison confirmed that order-aware energy recycling provides additional transmit energy for later scheduled WDs and helps support throughput-limited users. The results also indicate that the performance gain depends on three coupled factors: selecting a relay with a strong HAP link, reducing local source-to-relay collection overhead through backscatter, and using the transmission order to guide recyclable UL energy toward throughput-limited source WDs.

## Figures and Tables

**Table 1:** Comparison of the proposed scheme with representative backscatter-aided wireless-powered studies.

Work	Core Optimization Variables	Energy Model	Fairness Objective	Key Limitation
[16,17]	Beamforming, time allocation, and relay operation	RF energy harvesting with wireless energy beamforming	Throughput-oriented design	No source-order design
[18]	Time allocation, reflection coefficient, and transmit power	RF energy harvesting	Throughput-oriented design	Limited multi-source energy causality
[19,20]	Time and resource allocation for relay transmission	RF energy harvesting	Throughput-oriented design	No order-aware energy recycling
[21]	Offloading decision, time allocation, and transmit power	RF harvesting and computation energy consumption	Energy-efficiency/ computation-oriented design	MEC-oriented objective
[22]	Time and resource allocation for multi-backscatter transmission	RF energy harvesting with multi-backscatter access	Throughput-oriented design	No active-UL energy recycling
Proposed scheme	Source order, time allocation, and transmit power	RF harvesting with inter-source recycled energy	Max-min throughput fairness	Centralized block-level optimization

## Data Availability

The data will be made available on request.
